# One Means of Telling the Sex of a Pigeon

**Published:** 1897-03

**Authors:** Paul De Bart


### ABSTRACTS FROM FOREIGN JOURNALS.

### FRENCH.

UNDER THE DIRECTION OF ALEXANDER GLASS, V.S.,
PHILADELPHIA, PA.

ONE MEANS OF TELLING THE SEX OF A PIGEON. Hold the pigeon lightly in the hand and lay it on its side, allowing the feet to be free. After the bird becomes quiet, if it holds the tail up and the feathers together, it is a male; if the tail-feathers are spread out like a fan, it is a female. The bird can also be held by the wings, and in this position the shape of the tail is very characteristic.—*Paul de Bart.*

